# Professor Mayowa Owolabi, WFNR Regional Vice-President for Africa: Adapted Interview from the 12^th^ World Congress for NeuroRehabilitation (WCNR), Vienna, 2022

**DOI:** 10.25122/jml-2023-1012

**Published:** 2023-03

**Authors:** Stefana-Andrada Dobran, Alexandra Gherman

**Affiliations:** 1 RoNeuro Institute for Neurological Research and Diagnostic, Cluj-Napoca, Romania; 2 Sociology Department, Babes-Bolyai University, Cluj-Napoca, Romania

**Interviewer: Stefana-Andrada Dobran**

**Interviewee: Professor Mayowa Owolabi**

Professor Mayowa Owolabi is a Professor of Neurology and pioneer Director of the Centre for Genomic and Precision Medicine. He is also the lead Co-Chair of the Steering Committee of the World Stroke Organization - *Lancet* Commission on Stroke, and a Fellow of the Academy of Science (Nigeria), Fellow of the American Academy of Neurology, and Fellow of the Royal College of Physicians (London).

Professor Mayowa Owolabi is a globally recognized leader in the field of science, with expertise in neurology, cardiovascular diseases, neurorehabilitation, and community-based genomic epidemiology of stroke in Africa. Prof. Owolabi is the leader of several collaborative studies in the field of stroke, including the Stroke Investigative Research & Educational Network (SIREN) as Overall PI and Systematic Investigation of Blacks with Stroke using Genomics (SIBS Genomics) studies. These studies have yielded landmark publications, including the discovery of the association between Apolipoprotein-1 and lacunar stroke. He is leading the first GWAS of stroke in indigenous Africans. He is the pioneer Chair of the largest study of cardiovascular diseases in Africa (H3Africa-CVD working group with >55,000 subjects).

Professor Owolabi has authored over 350 publications in peer-reviewed journals and owns several inventions, including a stroke phenotyping software with a patent. He also serves as the Associate Editor of BMC Neurology and sits on the editorial board of many high-impact journals.

**S.A.D.:** Hello, dear Professor Owolabi! We are here, in Vienna, for the 12^th^ World Congress for NeuroRehabilitation, organised by the WFNR. What is your first-hand opinion of the event so far, and have you participated in any previous editions?

**M.O.:** I think it has been a wonderful experience, almost one week long, right from the pre-congress workshops, which I coordinated with Professor Thomas Platz. We had a book – Clinical Pathways in Stroke Rehabilitation: Evidence-based Clinical Practice Recommendations, which we used for the Teaching Course, and it was well attended and it was really empowering people through education by disseminating information, which can help people to acquire skills, even though remotely, those who participated in the course, they can take some exams and get some certification. But beyond that, all the plenary sessions have been extremely fantastic, looking into the future, innovations that are likely to transform the lives of people living with neurological disabilities across the globe. And this is extremely crucial – that this is happening this year, which has been a very, very remarkable year for neurology and, indeed, neurorehabilitation, all across the globe with the launching of the WHO Intersectoral Global Action Plan against Epilepsy and Other Neurological Conditions and also a lot of initiatives that were launched also at the level of the WHO this year – The Brain Health Initiative and something launched very recently, about two weeks ago, on disabilities. This is really very remarkable. The congresses – yes, I attended several, I had several attendances since 2008, every two years, with the Covid pandemic, there were issues, it was skipped, it was virtual, but this one is hybrid, and maybe subsequent will also be hybrid. But it has been very exciting meeting people again. WFNR is a very large family, and what our mission is, what we are trying to do is to make sure that people with neurological disabilities, who survived a neurological injury, can live a good life. What do we mean by ‘a good life’? We want to make sure that we add years to their life and that they live longer, but beyond that, we also want to add life to their years. We want them to have a good quality of life, but even beyond that, we want to add meaning to their life. We want people to live a meaningful, purposeful, productive life, regardless of the condition they might have.

**S.A.D.:** Thank you! And what do you believe is the overarching theme of this year’s congress?

**M.O.:** As always, I think, apart from neuro-technologies, which are emerging, where evidence is being built, I think one of the key themes of focus of this congress is, of course, about the interdisciplinary or transdisciplinary care, which is to be made available to all the people who need these, across the globe. That is, actually, the challenge. That’s the challenge we are trying to solve, putting our minds together in this sense.

**S.A.D.:** From your perspective, what is the role of hybrid multidisciplinary events in developing neurorehabilitation research and practice, and what other avenues are worth exploring?

**M.O.:** Neurorehabilitation, by its nature, is multidisciplinary, or better still, interdisciplinary, or best of all – **transdisciplinary**. Why is that? Many patients who have neurological disabilities do not have just one or two, some have multiple disabilities. And so, a team approach is required to address these issues and attend to the needs of the person as a whole - a holistic, humanistic approach that deals with all the issues. And they are all interrelated. For instance, if a stroke patient is depressed and is not motivated, and does not get psychotherapy to improve motivation, he may not participate in physiotherapy, in terms of voluntary activities that are required to enable him to have to improve in terms of the intensity, the duration and the quality of such practices. The same thing happens with speech and language therapy. And if a psychotherapist wants to attend to someone with aphasia without the help of a speech and language therapist, that may not work. So, it’s of necessity in a multidisciplinary team. And therefore, all interactions – in terms of teaching, in terms of service, in terms of education – all activities that have to do with neurorehabilitation have to be multidisciplinary. Now this can happen in the setting of a congress like this one, which could be completely virtual - seminars, webinars can be done also online, multidisciplinary courses, hybrid congresses like this one, and, of course, physical congresses. And then, in a more prolonged manner, in a more generalised manner, universities or faculties, or departments that train people along different career paths in a multidisciplinary setting. Like a faculty of neurorehabilitation, for instance, that has departments for speech therapy, or speech pathologies, and all the other disciplines. It is also important for it to be transdisciplinary, meaning that each person also needs to know a little bit about the role of the other people and when to call them, and then, they need to meet together for the goal setting to ensure that the patient actually improves along all the different spheres of the patient’s life. So, different possible settings.

**S.A.D.:** What are the most crucial factors that clinicians should consider in stroke neurorehabilitation?

**M.O.:** The most crucial factor that stroke physicians should consider is that rehabilitation is part of the continuum of care and is actually, perhaps, the most important. If the patient has survived, then what next? Is it the end of the life for the patient? Can the patient still go back to work? Can the patient participate in societal activities, can the patient really still lead a good life? That should be the concern of the stroke physician. And to accomplish this, he must make sure that he coordinates and leads a team-based approach that is involved in fully assessing all the impairments, deficits, and challenges with roles and participation of the individual, set goals, deliver multidisciplinary or transdisciplinary care, and assess and evaluate and make sure that the patient is getting better. Of course, as this is happening, the stroke physician must also pay attention to any comorbidities, pay attention to risk factors profiling and control to prevent the recurrence of stroke. So, all of these are important. In the end, the stroke patient then gets the best treatment and is able to recover as much as possible, and the stroke physician is happy along with his team.

**S.A.D.:** How can multidisciplinary stroke rehabilitation influence mortality and morbidity concerning this disease?

**M.O.:** Some deficits happen in patients with stroke, for instance, let me take a particular example, *hemiplegia*, which is a weakness or complete paralysis of one side of the body. If that is not taken care of and there is no recovery of function, that could lead to venous thrombosis, and that could lead to death. So, physiotherapy is very important to ensure that that is restored. Then, let’s take another one, which is *dysphagia*, which is also very common, meaning problems with swallowing. If that is not taken care of – in fact, in many parts of the world, it is the number 1 killer of our stroke patients, there will be aspiration pneumonia, and who is going to take care of that?! It is actually not the stroke physician, it’s the speech and language therapist. Multidisciplinary care is extremely important. And how do you take care of morbidity? You want to improve the quality of life; you want to reduce disabilities. If you don’t do this in a multidisciplinary setting, including the psychotherapists and everybody, and the patient is not seen as improving and is then having a sense of hopelessness, helplessness, and depression, that is morbidity. And if the patient is feeling useless or like a burden on other people, that is morbidity. Or if the patient is feeling impotent and impoverished, you know, no longer having any income, he can’t do what he wishes to do – for instance, someone who was a lecturer, a musician or a footballer, or enjoying some hobbies, just seeing himself not doing those things again, that is incredible morbidity. And sometimes, patients maybe even wish to die. But we can change the situation in this care setting that is transdisciplinary, that looks forward, sets goals, and gives hope, and then we can see incremental progress. And then we know that the future is bright.

**S.A.D.:** How can we improve evidence-based stroke guidelines at the international level?

**M.O.:** Fantastic question! I would like to start by saying that stroke rehabilitation compared to acute care, is not sufficiently based on the results of robust clinical trials. Meanwhile, it is extremely critical, as one of the major pillars of the stroke quadrangle; there are 4 pillars of the stroke quadrangle: surveillance, prevention, acute care, and rehabilitation, which is, actually, chronic care, which stays longer. But in terms of high-powered, multi-centre studies looking at innovations in terms of treatments, be it neuro-technologies, be it care settings, be it novel drug therapies that could modify outcome, neuroplasticity, there are very few and far between. The number of those participating in those studies and trials is very small, and so, it is very difficult to translate them across the globe. And in fact, I dare to say that, for neurorehabilitation of stroke compared to acute care, most of what we do, like 60-70% of what we do, is not based on a high grade of evidence in terms of systematic reviews or meta-analyses, or randomized control trials. Again, because designing this is very difficult and complex because of the different factors in the care setting and because they are also expensive, and they cannot be easily conducted across the globe. Also, because they’re expensive and because the resources may or may not be there. While these actually are extremely important to lay the foundation for RCTs […] with high-grade evidence based on recommendations and interventions, and solutions across the globe. It’s important to advocate and draw attention to the need to ask new questions, conduct new studies, and pragmatic trials that are adequately powered and multi-centre in order to bring those to the next phase for stroke rehabilitation or for rehabilitation in general, whereby we can make the limb to walk again, we can make the blind to see again, we can make the dumb to speak again. We can make people recover lost functions or aid them with coping strategies using neuro-technologies or using stem cell therapy or novel therapies so that, in the end, they can recover as many functions as possible. But that requires a lot of attention, a lot of resources, a lot of energy, and a lot of effort. Thank you very much!

**S.A.D.:** Thank you very much for the interview!

**M.O.:** Thank you!

